# Limitations of mRNA amplification from small-size cell samples

**DOI:** 10.1186/1471-2164-6-147

**Published:** 2005-10-27

**Authors:** Vigdis Nygaard, Marit Holden, Anders Løland, Mette Langaas, Ola Myklebost, Eivind Hovig

**Affiliations:** 1 Department of Tumor Biology, Institute for Cancer Research, The Norwegian Radium Hospital, Montebello, 0310 Oslo, Norway; 2 Norwegian Computing Center, P.O. Box 114 Blindern, 0314 Oslo, Norway; 3 Department of Mathematical Sciences, Norwegian University of Science and Technology, 7491 Trondheim, Norway

## Abstract

**Background:**

Global mRNA amplification has become a widely used approach to obtain gene expression profiles from limited material. An important concern is the reliable reflection of the starting material in the results obtained. This is especially important with extremely low quantities of input RNA where stochastic effects due to template dilution may be present. This aspect remains under-documented in the literature, as quantitative measures of data reliability are most often lacking. To address this issue, we examined the sensitivity levels of each transcript in 3 different cell sample sizes. ANOVA analysis was used to estimate the overall effects of reduced input RNA in our experimental design. In order to estimate the validity of decreasing sample sizes, we examined the sensitivity levels of each transcript by applying a novel model-based method, TransCount.

**Results:**

From expression data, TransCount provided estimates of absolute transcript concentrations in each examined sample. The results from TransCount were used to calculate the Pearson correlation coefficient between transcript concentrations for different sample sizes. The correlations were clearly transcript copy number dependent. A critical level was observed where stochastic fluctuations became significant. The analysis allowed us to pinpoint the gene specific number of transcript templates that defined the limit of reliability with respect to number of cells from that particular source. In the sample amplifying from 1000 cells, transcripts expressed with at least 121 transcripts/cell were statistically reliable and for 250 cells, the limit was 1806 transcripts/cell. Above these thresholds, correlation between our data sets was at acceptable values for reliable interpretation.

**Conclusion:**

These results imply that the reliability of any amplification experiment must be validated empirically to justify that any gene exists in sufficient quantity in the input material. This finding has important implications for any experiment where only extremely small samples such as single cell analyses or laser captured microdissected cells are available.

## Background

Standard protocols for microarray analysis are generally based on samples with more than 1–5 *μ*g of total RNA. However, there is an increasing interest in transcription profiling of small samples, as large amounts of material can be difficult, if not impossible, to obtain in both clinical and experimental settings. Fine needle aspirates (FNA) (~1–2 *μ*g) and fine needle core biopsies (~2 *μ*g of total RNA) offer feasible, atraumatic clinical sampling procedures of limited material. Advances in technology designed for selective collection of specialized cells such as laser capture microdissection (LCM), yields homogenous minute material for further analysis. Following standard protocols in a pilot study, Assersohn *et al*. [1] had to exclude 85% of the FNA of breast cancer samples from further microarray analysis due to insufficient material. The most common strategy to circumvent the large material requirement using a standard procedure has been to amplify the starting mRNA. The procedure for global mRNA amplification can be performed either linearly, using T7-based *in vitro *transcription [2-4] or exponentially, using a PCR-based amplification [5] or a combination of both [6]. These methods all include an initial reverse transcription. For the linear method, the yield is 10^3 ^to 10^6 ^fold amplification when using from one to three rounds of amplification, while an even higher fold up-scaling is possible using exponential amplification. Using either method one can technically amplify RNA from a single cell for gene expression analysis. The protocols for linear and exponential amplification have been subjected to optimizations [7-9], and a number of variations have been described, such as SMART-PCR [10] and Terminal continuation (TC) RNA amplification [11]. However, the most important issue for any amplification protocol to be used in combination with quantitative analysis of gene expression, is that the relative transcript abundance present in the initial mRNA sample is maintained throughout the procedure. In a previous study, we found that the gene expression ratios were not completely preserved between linearly amplified (from 200 ng total RNA) and non-amplified material [12]. Microarray experiments are subjected to variability from a range of sources, and under the experimental conditions applied, we showed that the impact of amplification was increased noise in the form of ratio distortions for some genes [12]. In this study we applied an ANOVA approach to examine the effects of variation as we reduced the amount of total RNA in the first round of aRNA production. We explored the lower range of RNA input that was technically feasible in our hands. A main concern is the lower sensitivity limits with respect to reliable data obtained from minute samples. This highly relevant issue remains under-documented in the literature. The presently published reports are focused on descriptive analysis of results from amplification from minute samples.

Furthermore, estimates of sensitivity limits have in these applications generally been defined as the minimum detectable abundance level that can be quantified in the respective protocols. Quantitative measures of reliability of the obtained data are most often lacking. In the two seminal papers first presenting detailed mRNA amplification protocols for use with microarray technology, the general consensus was a decrease in correlation coefficients and concordance when evaluating diminishing input RNA amounts [3,4]. Increased discrepancies between the data sets when comparing the input total RNA range of 200 ng, 10 ng and 2 ng against 10 ug input were observed [4]. Goley *et al*. [13] showed that serial dilution of RNA subjected to amplification resulted in increased dissimilarities when compared to unamplified RNA. Several studies have shown that starting with minute samples in the initial amplification round, the number of genes detected with sufficient signal for further analysis decreased [14-16]. Reducing the number of cells under investigation to approximately 1000 cells, Mohr *et al*. [16] observed that only 19% of the 9850 genes displayed a signal at least two times more than the background signal. A total of 79% of the genes detected had signals above background, indicating that the majority of genes were in the low to moderate expression range. For the examination of lower sensitivity levels, there are examples of studies that have applied spiked transcripts of known concentration [17,18]. Using a PCR based amplification strategy for single cell analysis, a limit of 80 copies per cell was reported to be the lower range for registration of two fold changes in input RNA [18].

For the purpose of applying mRNA amplification and small sized samples in future studies, we chose to define the lower sensitivity limit of an mRNA amplification protocol as the number of mRNA copies needed not only to be detectable but more importantly, produce reliable data. Unlike previous studies, our goal was to quantitatively analyze the reliability of expression data for each transcript present on our arrays as we reduced input RNA material and thus to deduce a transcript dependent cut-off limit with respect to reliability. We applied a novel model-based method, TransCount [19], that estimates absolute transcript concentrations from expression data for each examined sample. We observed the role of stochastic perturbations on the amplification of small number of reactant mRNA molecules. Stochastic noise dominating the signal in amplified mRNA analysis has not been acknowledged in previous reports. With the strategy of computing correlation coefficients between transcript concentrations for two different samples, the sensitivity limits of other amplification methods can also be validated. More importantly, the procedure provides a rational basis for the selection of genes that possess true predictive power.

## Results

### Descriptive statistics

**Figure 1:**
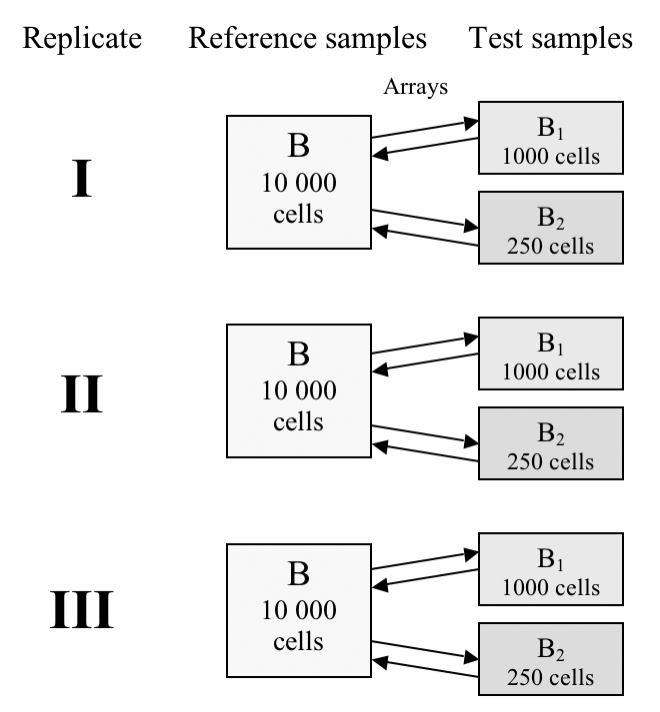
**Experimental design**. Three replicates of each cell size sample were amplified. Each reference replicate B (10 000 cells) was hybridized to test samples B_1 _(1000 cells) and B_2 _(250 cells), respectively, in a dye-swap strategy. The arrows represent arrays, alternating in direction to indicate dye-swap. In total, six arrays were used for each test sample size versus reference.

Total RNA was isolated from three aliquots of 10 000 MT-1 cells and amplified as undiluted reference material. Cells were diluted to obtain aliquots of 1000 and 250 test cell samples. Total RNA isolated from parallel aliquots of 10 000 cells not used in this study, yielded an average of 115 ng when measured using a Nano Chip assay on the Agilent Bioanalyzer. On the same Nano Chip, total RNA from 1000 cells was not detectable. We therefore used 115 ng from 10 000 cells as a reference for relevant RNA calculations in this paper. It is important to emphasize that these downstream RNA calculations, such as amplification factors, only provide estimates and do not reflect sampling errors present during cell aliquot preparations that have undoubtedly influenced the actual yields measured. Hence, we infer that the total RNA yield from 1000 and 250 HeLa cells is approximately 11,5 ng and 2,88 ng respectively (Table 1). In addition to RNA from the cells, all samples were spiked with synthetic RNA transcripts obtained from the Lucidea Universal Scorecard kit to monitor the amplification procedure and used for inference purposes to bootstrap the transcript concentration calculations. These transcripts also served as carriers throughout the multistep amplification procedure. The fraction of mRNA in the total RNA is in the range of 1–3% and by applying the theoretical average of 2% mRNA we have summarized the estimated mRNA input values for each cell size sample (Table 1). We calculated that the average fold amplification factor after two rounds in these three cell size samples (10 000, 1000 and 250) was 1.0 × 10^4^, 0.69 × 10^4 ^and 0.89 × 10^4^, respectively (Table 1). The purity of the aRNA samples was measured by absorbance readings. With the exception of one 250 cell batch (ratio = 1.8), the ratios obtained by absorbance measurements were between 2.0–2.6 for all samples. The size distribution of the aRNA products were visualized using an Agilent Bioanalyzer assay. The distribution peaked at approximately 500 bp for all samples (data not shown). Products from 1000 and 250 cells were not larger than 1000 bp, while for the reference sample of 10 000 cells, they were detectable up to 2000 bp. Distinctive peaks representing the most concentrated Scorecard transcripts could be observed on the graphs (graphs not shown). Using a dye swap strategy, we analyzed data from six hybridizations with 10 000 cells versus 1000 cells and six hybridizations with 10 000 cells versus 250 cells, making a total of twelve arrays (Figure 1). On average, 25% of the spots (n = 11791) were filtered from arrays hybridized with material amplified from 1000 cells in the test channel, compared to 37% of the genes filtered when amplified RNA from 250 cells was in the test channel. The data for each filtering per array is presented in Table 2. Examining the filtering of weak spots for each set of cell size samples separately, we found that the average number of signals scored was 20% and 33% less for 1000 and 250 cell samples, respectively, when the reference sample (10 000 cells) was set to 100% (data not shown). These figures are partially confounded by reduced target material labelled in the test samples.

**Table 1:** Estimates of input RNA quantities and resulting yield for 10 000 cells (reference) and 1000 and 250 cells (test), respectively. Calculation of the average fold yield of aRNA after two rounds of amplification are based on the assumption that 2% of total RNA represents mRNA.

Cell sample size	~Total RNA	~mRNA (2% of total RNA)	Synthetic mRNA	Sum mRNA	Average aRNA yield	Average amplification factor
10 000	~115 ng	~2.3 ng	3.0 ng	5.3 ng	54 *μ*g	1.02 × 10^4^
1000	~11.5 ng	~0.23 ng	0.3 ng	0.53 ng	3.66 *μ*g	0.69 × 10^4^
250	~2.88 ng	~0.058 ng	0.075 ng	0.133 ng	1.18 *μ*g	0.89 × 10^4^

**Table 2:** Filtering per array.

	Arrays hybridized with sample size 1000 cells							Arrays hybridized with sample size 250 cells						
Array number	2	12	1	3	4	11	mean (SD)	6	9	7	5	8	10	mean (SD)
Genes flagged by Genepix	1189	1188	2344	1105	1272	456	1259 (556.2)	1731	1726	1155	1009	4466	3798	2314 (1327)
	10.08%	10.08%	19.98%	9.37%	10.79%	3.87%	10.7%	14.68%	14.64%	9.80%	8.56%	37.88%	32.21%	19.6%
Genes flagged manually	10	17	42	27	21	51	28 (14.3)	21	11	104	359	29	69	98.8 (120.6)
	0.08%	0.14%	0.36%	0.23%	0.18%	0.43%	0.2%	0.18%	0.09%	0.88%	3.04%	0.25%	0.59%	0.8%
Additionally filtered by spot-background>2× standard deviation of background	113	261	3459	2455	2229	1390	1651 (1198)	1602	562	2020	1292	3886	2184	1924 (1023)
	0.01%	2.21%	29.34%	20.82%	18.90%	11.79%	13.9%	13.59%	4.77%	17.13%	10.96%	32.96%	18.52%	16.3%
Sum	1312	1466	5934	3587	3522	1897	2953 (1614)	3354	2299	3279	2660	8381	6051	4337 (2173)
	10.17%	12.43%	49.68%	30.42%	29.87%	16.09%	24.8%	28.45%	19.50%	27.81%	22.56%	71.09%	51.32%	36.8%

### Array quality index

The potential problem of using inadequate or poor quality input RNA in the test channel is relatively uniform signal intensities because of low fluorescence signals. In such cases the reference channel, which is normally designed to provide a large dynamic range of consistent signal intensities, will be the driving force of the ratio calculations and hence there will be a correlation between gene expression ratios and the signal intensities of the reference channel. To examine this potential problem we used two parameters of array quality index as described by Assersohn *et al*., [1]. We first examined the SD of the log 10-based signal intensities in the test channel (material from 1000 or 250 cell samples) as a measure of the dynamic range. The span was between 0.59 – 0.3 for all the arrays (Table 3). For 1000 and 250 cell samples the mean value was 0.48 and 0.38 respectively. A defined minimum threshold value for the SD of the signal intensities was not firmly established in the study by Asserhohn *et al*., [1]. However they applied 0.25 as a minimum requirement. In a preliminary test hybridization, we used 0.2 *μ*g aRNA diluted from one of the reference samples as target material in one of the channels and for comparison, we calculated the SD of the signal intensities for this channel to be 0.5. In the twelve arrays analyzed in this study, the SD's of the signal intensities obtained from the test channel were comparable or higher (0.45 – 0.59) in 50% of the hybridizations. No array had a SD lower 0.3. Hence, we found these magnitudes to be sufficient for the arrays to be used in further analysis and verified by calculating the correlation between log_10_-ratios and the log_10_-intensities of signals in the reference channel to ensure that the gene expression ratios were not dominated by the reference target which was prepared from a larger amount of input RNA. The correlations calculated for the twelve arrays were in the order of 0.13 – 0.48, with a mean value of 0.24 and 0.33 for the 1000 and 250 cell samples respectively (Table 3). In comparison, the correlation obtained from two self -self experiments with either even and uneven target preparation in a pilot study was 0.01 and 0.117 respectively. Hence, due to lack of evident correlation between gene expression ratios and reference channel signal intensities, we can exclude the potential domination of the reference channel due to uneven targeting on the arrays used in this study.

### Multiple hypothesis testing

From these self-self experiments, the expected log_2_-ratio values from the processed data were 0 for each gene in every microarray experiment, assuming no influence of material quantity in the initial steps of the amplification. However, the reduced number of genes detected above background levels indicated that low signals corresponding to moderate or low-copy transcripts were potentially more affected by background noise and thus less likely to correlate when performing expression level comparisons to a reference. In our previous report, we found that transcript dependent bias was not apparent in the optimal starting amount (200 ng total RNA) of the amplification protocol [12]. However, by drastically reducing input RNA in the amplification reaction, we observed in this study that the levels of detection were altered and variability introduced. To assess the observed variability in gene expression ratios, we used multiple hypothesis testing to identify genes with log_2_-ratios statistically significantly different from 0. The statistical analyses were based on filtered, transformed, normalized and dye-swap averaged data using a moderated t-test. P-values were calculated based on empirical Bayes t-test [20]. For the 10 000 versus 1000 cells experiments, we estimated the proportion of genes not differentially expressed to be 66.3%. Using a false discovery rate (FDR) cut-off of 0.05, we found 179 features (1.8%) that were significantly differentially expressed. For the 10 000 versus 250 cells experiments we estimated the proportion of genes not differentially expressed to be 57.1% and a FDR cut-off of 0.05 resulted in a list of 639 features (6.1%) that were statistically significant differential gene expression. Interestingly, between the two sets of significantly differentially expressed genes, there were 110 features in common. We found no connection between these 110 features with respect to signal intensity range or length of probe on array. We extracted the estimated transcript concentrations for these features and found the values for 82 features, representing 70 genes, as some were printed in duplicates on the array. As expected there was a large concentration span. For the 10 most concentrated genes, with the exception of one gene, the high transcript abundance was not maintained in the test samples compared to the reference samples as deduced from the respective raw data. For the remaining 60 genes, the general trend was equal or higher signal intensities in the test samples with respect to the reference channel. After normalization, these genes were overexpressed in the test samples, and hence, significantly differentially expressed.

### ANOVA analysis

We assumed that the variance observed between test samples and the reference was due to limited material in the test samples. To investigate this assumption we identified all potential factors contributing to variation and constructed an ANOVA model to estimate their importance, and thus isolate the effect of reduced starting material. The estimated contributions from the sources of variation modelled are presented in Table 4. We noted that *μ*, C, D and A were small as expected since the ratios are normalized. The dye-gene (DG) interaction was of moderate size, possibly as a result of difference in amount labelled in the two channels. The cell sample size-gene (CG) interaction was small, indicating that the noise due to differences between sample size 1000 and 250 cells was quite small. In accordance with the results from multiple hypothesis testing, the gene effect (G) was relatively high. For the interaction between replicates and gene (BG, B_1_G, B_2_G), we obtained the gradient of noise level in the expected order, from the lowest in 10 000 cells to the highest in 250 cells. There was a relatively small difference between the 1000 and 250 replicates.

**Table 4:** Parameter estimates in the ANOVA model. This is a mixed-effects model, as the first three effects are fixed and the others are random. The noise in these experiments was largely due to gene and to the interaction between replicates and gene. Reduction in samples size yielded increased noise since

Fixed Effect	Explanation	Estimated value
*μ*	Fixed overall level	-0.13
C	Sample size; 1000 or 250 cells	0.055
D	Dye ratio; cy3/cy5 or cy5/cy3	0.10
Random effects E~N(0, *σ*^2^_E_)	Explanation	Estimated standard deviation ()
A	Array; 1,...,12	0.14
G	Gene; 1,...10643	0.47
CG	Interaction: cell size sample and gene	0.044
BG	Interaction: replicate (10 000 cells) and gene	0.17
B_1_G	Interaction: replicate (1000 cells) and gene	0.41
B_2_G	Interaction: replicate (250 cells) and gene	0.45
DG	Interaction: dye ratio and gene	0.18
*ε*	Model and measurement error	0.33

### Absolute transcript concentration estimates and cut-off limits for unreliable data

**Figure 2:**
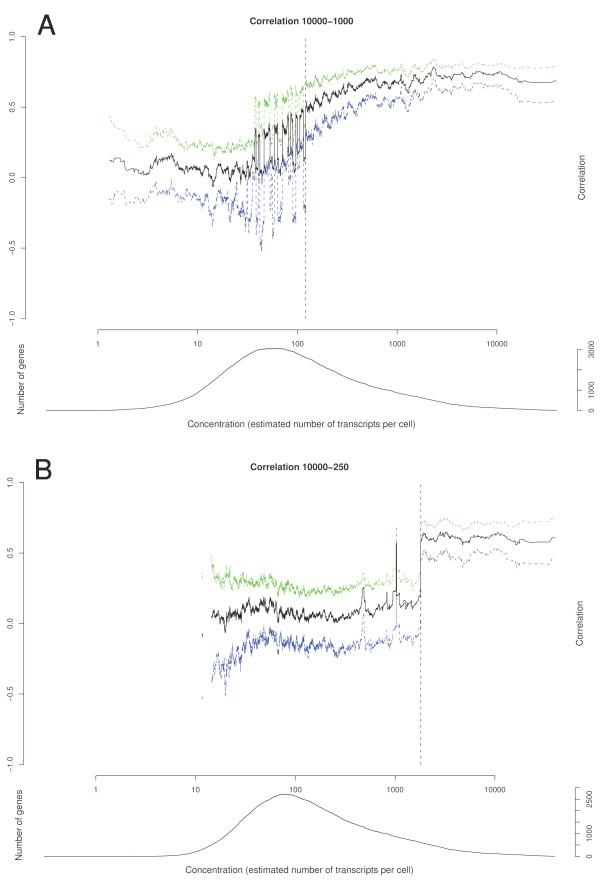
**a and b. Correlation of transcript concentration estimates**. Using the TransCount method, we obtained for each gene the distribution of the correlation coefficients between the reference sample transcript concentrations and the 1000 (250) cell sample transcript concentrations. Summary values for a certain gene in the (mean) 1000 cell samples versus the (mean) reference samples, are plotted against the estimated concentration for the (mean) reference sample in Fig. 2a. The black solid line is the median correlation coefficient values. The blue and green dashed lines are the 2.5% and 97.5% quantiles, respectively. The vertical dashed black line is the reliability threshold 121, i.e. the value for which the probability of positive correlation is at least 0.99. Similarly, information about the distribution for the (mean) 250 cell sample and (mean) reference is summarized in Fig 2b. In this case, the reliability threshold is 1806. The number of genes per concentration is shown below each respective plot. For a certain concentration c, the number of genes is counted from the interval

Larger replicate-gene (B_1_G and B_2_G) interactions obtained from the ANOVA analysis imply a greater degree of unreliable data present in the data set obtained from reduced samples. To strengthen possible biological findings in small samples, it is of great value to remove uncertain measurements. Empirically, observations suggested that these measurements were characterized by low signals related to low transcript abundance. We applied the TransCount model as an approach to eliminate unreliable data based on quantitative measures of template input in the initial amplification and for the threshold determination. With TransCount we found estimates of transcript concentrations for each gene per sample. For the reference (10 000 cells), a conversion factor was calculated for obtaining absolute concentrations. The conversion factor was generated by first estimating the concentrations estimates of the synthetic Scorecard templates, and secondly, deducing the factor by using linear regression. The absolute concentrations of each of 8116 genes present in the reference sample (10 000 cells) were estimated. For values above 0 (n = 8085), the range was between 0.3 and 40 000 transcripts per gene in an MT-1 cell. These quantities represent the contents of one cell prior to amplification. Multiplying by 10 000 cells, the amplification factor and the fraction of aRNA used for labeling, the range was between 5.8 × 10^5 ^and 7.8 × 10^10 ^transcripts per gene in the labelled cDNA pool that was applied to the array and represented the reference channel (Table 5). For this calculation, we assumed efficient conversion from aRNA to cDNA. Using TransCount, we obtained, in addition to the transcript concentrations for each gene and sample, the posterior joint probability distribution of all concentrations. This distribution could be used for computing various distributions and probabilities. We used it for calculating the distribution of the Pearson correlation coefficient between the reference sample concentrations and the 1000 cell sample concentrations for each gene (Fig. 2a). Similarly, the same procedure was performed for the reference and 250 cell samples (Fig. 2b). The graphs clearly showed that the correlation coefficients were copy number dependent. High copy numbers yielded high correlation coefficients. As expected, fewer transcripts per gene were necessary to obtain high correlations among genes amplified from 1000 cells compared to genes amplified from 250 cells. In Fig. 2a we observed a region where certain molecules were influenced by stochastic effects resulting in correlation coefficients alternating between poor and good. To define a cut-off value that ensured the exclusion of unreliable data in downstream analysis, we calculated the probability of positive correlation for each gene based on the distribution of the correlation coefficients. For the 1000 (250) cell sample, the probability of positive correlation was at least 0.99 when concentrations were 120.881 (1806.214) or more. Hence, in experiments using 1000 cells, the least number of templates required for further analysis was 121 copies per cell. For 250 cells the threshold was 1806 copies per cell. To convert the limit from minimum transcripts per gene in a cell to minimum number of transcripts per gene in the cDNA pool applied to the array, we multiplied by cell sample size, amplification factor and fraction of aRNA in the labeling reaction (Table 5). This calculated threshold was six times higher when initially starting from 250 cells compared to 1000 cells. With respect to the serially diluted synthetic templates, the two most diluted calibration spikes (cYIR09 and cYIR10) in the samples with 1000 cells were below the threshold, while for 250 cell samples, the five most diluted calibration spikes (cYIR06-cYIR10) were below threshold. The number of genes left for analysis when applying the cut-off values 121- and 1806 copies per cell were 3149 and 390, respectively. This represented an eight times difference of data loss when the sample was reduced from 1000 to 250 cells. Hence, for 250 cells as input value in the amplification procedure, only a relatively small fraction of the genes queried on the array were suitable for further functional analysis.

**Table 5:** Conversion of gene transcripts per cell to gene transcripts applied to the array. The number of molecules per gene hybridized to the array varied between 5.8 × 10^5 ^– 7.8 × 10^10 ^with respect to the reference sample. The reliability threshold in terms of minimum number of molecules per gene applied to arrays for 250 cells, 6.8 × 10^8^, was six times higher than the threshold for 1000 cells.

Cell sample size	Description	Copies per cell	Amplification factor	Fraction aRNA labeled	Equivalent number of molecules applied to array
10 000 reference	transcript concentration range	0.3 – 40 000	1.02 × 10^4^	0.019	5.8 × 10^5 ^– 8.1 × 10^10^
1000	reliability threshold	121	0.69 × 10^4^	0.137	1.1 × 10^8^
250	reliability threshold	1806	0.89 × 10^4^	0.169	6.8 × 10^8^

**Figure 3:**
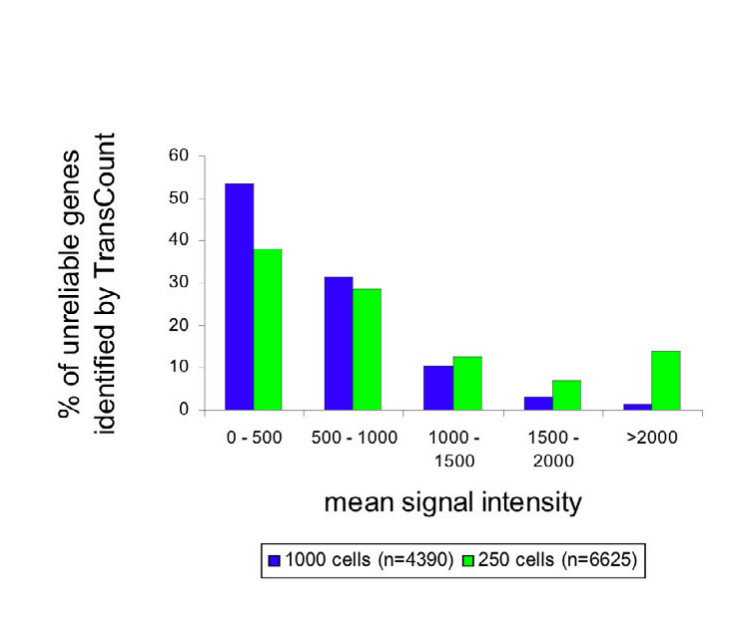
**Signal intensity distribution of genes below the statistically defined threshold**. Accumulation of genes in the low to moderate detection range when examining the mean signal intensity distribution of genes below the reliable threshold, but not filtered by the weak spot criteria. These genes were observed in at least two of the three dye-swap duplicates.

We examined the signal intensity distribution of the genes that were filtered according to the cut-off value, but not by the weak spot filter criteria used when pre-processing the data. We chose to investigate the genes with at least two observations out of the three dye-swap duplicates. As expected, the majority were in the low signal range, with a slightly wider distribution range for data obtained from 250 cells (Fig. 3). A filtering criterion of <1500 in mean signal intensity would remove 95% of the genes categorized as unreliable by TransCount when amplifying from 1000 cells. This intensity criterion was chosen by visual inspection of Fig. 3. However, due to the greater spread of intensities in the 250 cell samples, a similar criterion was more difficult to determine. A removal of 95% of the genes required a signal >3270.

## Discussion

To examine the conservation of ratio profiles between increasingly smaller cell size samples, we performed a series of analyses on microarray data generated from an experimental design using one source of material, but with varying amounts RNA into the amplification procedure prior to hybridization. Many studies have applied serially diluted RNA from the same total RNA sample to evaluate amplification procedures [3-5,13]. However, this does not approximate experimental conditions for separately processing minute samples from start to finish, as is the case in patient sample handling. Hence, the experimental procedure started with isolation of total RNA from 9 independent and cell counted samples drawn from the same cell culture. As the samples varied in size, two RNA isolation kits were applied that each were specially designed to cover a certain quantification range of cells. Both kits originated from the same manufacturer. They were based on the same chemical components and only differing in the RNA binding capacity of the silica fiber matrix and subsequent elution volume. Our aim was to maximize total RNA yield as suboptimal RNA isolation from the small test samples could result in biased data. In order to optimize the generation of amplified products and the degree of correlation between parallel samples, we thus chose to include the entire sample in the amplification procedure setup. We made conservative estimates of amplification factors, knowing that they are prone to sampling errors in the actual number of initial cells in each sample, in addition to variable efficiency in isolation of total RNA. For the lower limit of input material, the sample size was set to 250 cells, as this is, in our experience, the lower range of the amplification protocol with respect to generating sufficient aRNA for labeling probes in a dye-swap strategy. The aRNA yields from the small test samples were a determining factor for the amount used in the target labeling reactions. We applied a relatively equal percentage of the aRNA yield from the two test samples sizes for the respective labeling reactions. The labeling amount for the reference target was set to a constant value across all arrays and thus uneven target amounts were used being aware of the confounding contribution to the data filtering process. It is clear that in realistic clinical settings, patient sample size variability may be extensive both between patients and within the same patient and obtaining sufficient aRNA to perform microarray analysis from particular samples may be challenging. The essential requirement for inclusion of scarce aRNA material in data analysis is sufficient array quality. We used array quality index parameters to ensure adequate quality of the target used in the test sample channel and lack of reference channel domination of the gene expression ratios (Table 3). An alternative approach to further increase the yield of scarce material in an attempt to equate reference target material amount in the desired number of hybridizations, would be to introduce a third round of amplification. Scherer *et al*. [21] reported that an additional third round only had a modest effect on reproducibility. However, a third round would increase the work load, as it is optimal to compare aRNA based on equal number of amplification rounds, and also risk plateau effects with high copy gene saturation especially if using a reference sample in the experimental design. In fact, two rounds of amplification sufficiently upscaled many genes to saturation levels in the reference samples used in this study. Extending the *in vitro *transcription step is not a recommended alternative for increasing aRNA amounts due to increase in non-template based products. *In vitro *transcription past four hours has been shown to have negative effects in that the aRNA becomes degraded and thus reduces the quality of post-amplification procedures such as microarray analysis [22].

**Table 3:** Array quality index. The SD of the log_10_-intensities in channel 1 give an indication of the dynamic range obtained from the test samples (1000 or 250 cell samples). The correlation coefficients between gene expression log_10_-ratios of the experiment and log_10_-intensities of channel 2 (reference sample) were calculated to confirm that the gene expression ratios are not determined by the signal intensities of the reference channel.

	Arrays hybridized with sample size 1000 cells							Arrays hybridized with sample size 250 cells						
Array number	2	12	1	3	4	11	mean	6	9	7	5	8	10	mean
Standard deviation of test channel signal intensity	0.6	0.49	0.3	0.39	0.52	0.59	0.48	0.45	0.53	0.3	0.34	0.3	0.38	0.38
Correlation ratio vs. reference channel signal intensities	0.13	0.23	0.35	0.29	0.26	0.2	0.24	0.27	0.28	0.26	0.25	0.48	0.43	0.33

The amplification protocol we used applies to cDNA arrays and modifications, as alternative approaches are required for oligo arrays due to the antisense orientation of the aRNA products.

Descriptive analyses of the twelve arrays showed a reduction of the number of genes with sufficient expression levels for further analysis in the test channel representing the aRNA generated from 1000 or 250 cells, compared to the reference channel. However, the average number of features scored on arrays with material from 250 cells (6300 genes) was comparable to the amount of data obtained using 30 *μ*g of unamplified total RNA (6400 genes) from the same cell source on comparable array prints. Loss of data and lowered number of genes detected in earlier reports indicate that there is a bias for inaccurate representation of low copy number genes in the amplified pool of aRNA from minute samples. Another important issue is that low signals corresponding to moderate or low-copy transcripts may be more affected by background noise and thus less likely to correlate when performing comparisons. Although candidate genes may be extracted, the low signal-to-background ratio indicates that investigators need to be aware of the potential for inconsistent data interpretation.

To study the proportion of inconsistent data when using amplified material, correlation coefficient calculations have been the main choice of statistically based analyses. This is especially true for estimations of inconsistency in data generated from amplified material compared to non-amplified or from different amplification protocols [13,23-26], However, more sophisticated statistical analytical methods have also appeared [27-30]. To analyze the fidelity that the amplification procedure has on differential gene expression between two different samples, a comparison of t-scores or posterior distribution of fold change for individual genes have been applied [28-30]. The reported results from these analyses were restricted to a small subset of genes, more specifically the outliers, e.g. the top ranked 10 genes or genes with a fold change of >2. Without generating specific gene subsets, we used a t-test in our previous study to indicate the preservation of ratio when amplifying the input material and illustrated by plotting the ratio difference vs. the p-value for each gene. Likewise, a similar approach was used by Schlingemann *et al*., [31] to show how ratio preservation was maintained when serially diluting the RNA amount used in their sense-oriented amplification protocol. For the purpose of our investigation in this study, we globally estimated the portion of genes differentially expressed in the reference and test samples based on p-values calculated from a moderated t-test. Using the same sample source, the expected log_2_-ratio value was 0. We found that the number of differently expressed genes increased when comparing the smallest test sample size (250 cells) with the reference. The increase in differentially expressed genes indicated increased variability when reducing sample size.

In this study we applied an ANOVA model to estimate how much variability was introduced due to technical aspects when amplifying from very limited material. Ideally, variable sample size should not affect ratio measurement uncertainties. However, we showed there was a non-negligible increase in variation when sample size decreased. In this study, the variation was captured by the interaction between replicate sample sizes of 1000 cells (250 cells) and gene, BG. This estimate was of considerable proportion with respect to the various sources of noise in the ANOVA model. We found it necessary to address, characterize and filter data affected by this variation.

The main factors possibly influencing loss and variance of gene expression data when reducing input RNA are sequence-dependent bias and transcript abundance bias. In our previous work we found no statistical evidence to support sequence dependent bias [12]. In exponential amplification methods, the situation may be different as DNA polymerase is more prone to sequence bias than RNA polymerase. Transcript abundance bias was not evident in the optimal starting amount (200 ng total RNA) of the amplification protocol and we found the degree of transcript representation in the resulting aRNA pool with respect to the initial total RNA transcript population to be satisfactory for the use in gene expression analysis. We also observed that amplification prior to microarray hybridization increased the data output when comparing to standard total RNA labeling and hybridization. This was due to improved signal intensities of genes with low transcript numbers. Hence, we were able to extract information from a substantially larger number of genes using amplified material compared to non-amplified. However, by drastically reducing RNA in the reaction we are initiating amplification from samples with fewer copies of rare transcripts and this may increase the chance of data loss and variation due to stochastic distribution of low copy number templates. We may classify mRNA transcript abundance into 3 groups; high, moderate and low abundance. There are about 12 000 different transcripts per cell and over 90% of these are represented by the low-copy abundance class, having 1–15 copies of the transcript per cell [32]. According to SAGE data, more than 83% of transcript were present in one copy per cell [33]. In an approach to examine the effects of abundance in a comparison between amplified and non-amplified data and replicates within the two groups, Sheidl *et al*. [34] divided the genes into 10 groups based on their intensity and Pearson correlation coefficients were calculated for each group respectively. The results demonstrated a clear effect of abundance bias on the correlation. For low copy number transcripts, the coefficient approached 0.2 as intensity reached background levels. The high variability in the low abundance region of both amplified and non-amplified experiments show that this is in an unreliable region of transcript concentration irrespective of the inclusion of an amplification step [34]. A point of relevance for comparative microarray studies can be drawn from a previous assessment of stochastic effect in quantitative PCR [35,36]. When the queried template is present in low copy numbers in both the test and reference sample, the variation in ratio between the two templates is dependent on the stochastic distribution of both transcripts, resulting in a multiplication of stochastic effects. These observations can further be related to the term Monte Carlo effect coined by Karrer *et al*. [37]. The Monte Carlo effect is described by small, random and template concentration dependent differences in amplification efficiency. Each template has a certain probability of being amplified or lost, resulting in inconsistent detection. The lower the abundance of any template, the smaller the probability is that its true abundance will be maintained in the amplified product. It is therefore evident that the role of stochastic effects on the amplification of small number of mRNA molecules needs to be recognized. These effects have not previously been identified in relation to mRNA amplification procedures and subsequent analysis. Gene expression measurements affected by stochastic fluctuations may result in highly misleading quantitative information. It is of great advantage to the investigator to be able to define the limit of accuracy in terms of the number of template copies needed to avoid such obscured gene expression measurements. Data acquired above this limit should theoretically be sufficiently reproducible for further biological interpretation. In a study addressing microarray sensitivity using non-amplified material, the minimum transcript concentration that provided reliable measurements was defined to be the interception between the distribution of signal intensities, with the distribution of signal intensities from differentially expressed genes [38]. As mentioned above, to analyze fidelity of amplification on differential gene expression between two different samples, comparisons of t-scores or posterior distributions of fold change for individual genes have more recently been applied [28-30]. However, reports from the analyses were in these cases restricted to a small subset of genes. Maintenance of fidelity across levels of gene expression was not investigated in these studies. Until now, few studies have presented quantitative strategies to establish the estimated number of transcripts necessary prior to amplification to consistently maintain the relative abundance present in the un-amplified sample. The use of spiked control transcripts of known concentration is one method to approach this question. Using a PCR based amplification strategy for the analysis of single cells, a limit of 80 copies per cell was reported [18]. For two rounds of PCR-based amplification of mRNA and subsequent single cell transcript profiling, the analysis of spike controls in a single channel experiment led to a cut off limit corresponding to 1–2 template copies per cell [39]. Fifty cycles were used in the first PCR round followed by thirty in the second. However, the use of two rounds of PCR is to our knowledge limited. Another approach is to verify and compare relative expression levels obtained with microarray data with other quantitative technologies to possibly set a cut-off limit. Quantitative RT-PCR is a commonly applied technique. However, verification is performed on a gene-by-gene basis and is not suited for a high throughput verification of low expressing genes. Neither is fluorescent correlationspectrometry (FCS), a direct and sensitive technology for quantification of single molecule (gene) in solution [40]. A medium throughput technique is the standardization of competitiveRT-PCR (StaRT-PCR) [41]. This multiplex PCR technique allowed the simultaneous expression and quantification measurements of 25 genes. However, the method requires careful design of competitive templates for each gene. Other emerging PCR based solutions, such as SmartProbe [42], where pre-designed primers and probes may be used to provide quantitative information on gene expression in a more high-throughput format. To avoid the obstacles inherent in most of the technologies mentioned above, and given the limited sample material, we propose the use of high throughput absolute quantification of the respective genes and a subsequent comparison with correlation coefficients obtained in our experimental design for establishment of a limit of accuracy for the sample cell size in question. The first step requires acquisition of absolute abundance data for the genes to be queried in the material source. Other high throughput methods for quantification of gene expression levels other than cDNA and oligonucleotide arrays are represented by techniques such as serial expression of gene expression (SAGE) [43] and massive parallel signature sequencing (MPSS) [44]. The advantage of SAGE is that it provides absolute quantification of transcripts compared to relative quantification using microarrays. The disadvantage with SAGE is the large number of clones that must be sequenced and the method is not as widespread as microarray technology. MPSS also allows a direct quantification like SAGE. In the MPSS procedure, the sequencing step is modified, such that parallel sequencing is performed directly on the solid bead. However, the technology requires special equipment and is only available through Lynx Genetics. In this study, we have presented the successful use of TransCount as a high throughput alternative approach for absolute transcript concentration per gene queried on the array. TransCount can handle experiments based on amplified as well as non-amplified material. The results of this study are based on cDNA array data but the model is also applicable to data obtained with oligoarrays.

To characterize the genes affected by variation, we ranked the genes in the data set according to estimated concentration of the transcripts in the reference sample (10 000 cells) and plotted the correlation coefficient on the y-axis. The plots clearly showed that the degree of variability was dependent on the template concentrations. This is comparable with the intensity-based analyses by Sheidl *et al*. [34]. To reduce variability and to correctly assess low abundance expression ratios, they suggest replicates of the experiment. Replicated hybridizations are not always an option when scarce material is applied, and although some measurements are strengthened by more observations such as those in the vicinity of background levels, uncertainties will still revolve around others, especially those affected by stochastic fluctuations. We proposed a filtering strategy based on the results of TransCount. In a sample consisting of 1000 cells, we found that at least 121 copies of a gene had to be present per cell for reliable preservation of expression level and subsequent detection. Likewise, the threshold was 1806 per cell when only 250 cells were used. In total, these thresholds amount to 121 000 and 451 500 templates per sample size investigated. This is a difference of 3.7 fold, which is comparable to the dilution factor of 4. The majority of unreliable genes was rejected if a minimum signal intensity filter of 1500 was required in the hybridizations where 1000 cells were used (see Fig. 3). In other words, unreliable genes are present in the moderate signal intensity range that generally is not considered to be a high variance range, as these signals are well distinguished from background levels. A similar minimum signal requirement was not easily determined for 250 cells as unreliable genes were not only low/moderately expressed, but also extended towards high expression. The reliability limit set by TransCount for the respective sample sizes was therefore not potentially influenced by unbalanced, aRNA target preparation, as large portions of the unreliable genes were sufficiently above background levels indicating that the threshold is in fact determined during the initial phase of the amplification procedure when mRNA templates are copied into cDNA. This fact applies to all other amplification protocols, even those with higher amplification yields than the one applied in this study. Further, this emphasizes the need for an alternative approach to remove biologically irrelevant data such as the one presented in this article where stochastic effects were acknowledged and minimum limit for reliable detection was devoid of such effects. Identification of which and how many genes were available for analysis if the sample size was further reduced was difficult to estimate with certainty given the limited number of samples sizes in our experimental design. However, assuming a linear trend, we can predict that for a sample size of 100 cells, at least 1 210 000 templates per gene to be monitored have to be present in the pre-amplification mixture for reliable measurements. This figure translates to 12 100 copies per cell, and for our particular RNA source, we are left with 35 genes for analysis. In this range, we should consider what is the reasonable minimum number of cells from which we can obtain any informative qualitative results even if only studying high expression genes. If our genes of interest are below the threshold, then microarray analysis will in fact not bear relevant information. The cut-off value, the number and identification of genes left for downstream analyses will vary depending on RNA source. It is evident that single cell analysis is beyond the sensitivity of this assay.

## Conclusion

For application of microarray technology combined with amplification of mRNA from nano- to picograms of mRNA, there is a pitfall of conferring biological relevance to unreliable data due to transcript abundant bias alteration of true gene expression. We demonstrated that with our strategy, and the use of TransCount, we can define limits with respect to number of transcripts necessary for meaningful interpretation of expression data from conditions using reduced input RNA, thus avoiding obscured expression variability. As the input cell number decreased, the necessary number of transcripts per cell increased. The potential impact of this should be reflected in the design of future studies. Assembling transcription concentration data from the source material of interest using TransCount should allow a prediction of which genes will provide meaningful results when minute samples of the same source are applied.

Using the framework presented in this study also allows evaluation of alternative amplification protocols, such as various PCR-based strategies

Furthermore, based on our findings regarding the sensitivity limit of our amplification protocol in use, only moderate to high expressing genes can be regarded in experiments with <1000 cells. All data from low expressing genes should be disregarded due to inaccuracy. For minute samples, any template diluted past a certain threshold copy number determined by assay sensitivity, will experience large variation in amplification whose origin is the stochastic nature of the biochemical reaction. In the event that the gene of interest is subjected to abundance dependent bias variation, only qualitative information can be obtained. In agreement with Stenman and Orpana [35], we conclude that quantitative expression profiling of single cells is only potentially possible for genes expressed at high levels that show reproducible expression in replicate experiments. Single cell analyses through mRNA amplification are burdened by stochasticity not only in the procedure itself, but also at the level of transcription as intrinsic noise [45], thereby severely limiting the precision of gene expression measurements. It is evident that sophisticated technologies for the selective collection of specialized cells are a step ahead of commonly available, high throughput quantitative expression profiling technologies. Caution is warranted when extrapolating biological relevance from the increasing number of expression profiling results published based on extremely low cell numbers.

## Methods

### cDNA arrays

The 13 k human cDNA arrays applied in this study were printed in house with cDNA clones from the Research Genetics 40 k cDNA clone library (Invitrogen, Carlsbad, CA.). The cDNAs were prepared from the clone library by PCR using M13 universal primers. The purified PCR products were resuspended in 3 × SSC and spotted on amino silane coated slides (CMT GAPS, Corning Life Sciences, Corning, NY) using a Micro Grid II robotic printer (Bio Robotics, Cambridge, UK). After printing the slides were cross-linked by UV to immobilize the double stranded probes. For details on the arrays, we refer to the website for the microarray core facility at The Norwegian Radium Hospital [46].

### RNA purification

The human carcinoma cell line, HeLa, was used throughout the study. The cells were maintained in RPMI media (Bio Whittaker Europe) supplemented with 10% calf serum (PAA Laboratories, Linz, Austria). Phosphate-buffered saline (PBS) was used to dilute detached cells from one 25 cm^2 ^culture flask and make aliquots of 10 000, 1000 or 250 cells. The cells were stored in lysis buffer (Stratagene, La Jolla, CA) at -70°C. Total RNA from 10 000 cells aliquots were isolated using Microprep kit (Stratagene), while the Nanoprep kit (Stratagene) was used for isolation of total RNA from 1000 and 250 cell samples. The eluted RNA was immediately applied in the subsequent amplification procedure.

### RNA amplification

Two rounds of RNA amplification were carried out as described earlier [12]. For the 1000 and 250 cell samples, all volumes in the amplification procedure were reduced by a factor of 0.5 with the exception of *in vitro *transcription reactions. They were performed in the standard volume quantity suggested by the manufacturer. The reason for decreasing the reaction volumes was to reduce the difference in the primer to template ratio and thus minimize possible production of primer-dependent products. Briefly, the eluted total RNA was speed vacuumed with a primer annealing mix consisting of a dT/T7 primer, synthetic reference RNA (Lucidea Universal Scorecard reference RNA, Amersham Pharmacia Biotech AB, Uppsala, Sweden) in the case of 10 000 cell samples, and synthetic test RNA (Lucidea Universal Scorecard test RNA, Amersham Pharmacia Biotech AB) plus 40 ng of tRNA in the 1000 and 250 samples. Both reference and test Scorecard RNA contained one set of calibration control templates serially diluted (cYIR01-cYIR10), and one set of ratio control templates (rYIR1-rYIR8). We diluted 3 × 1 *μ*l of scorecard RNA by the following scheme: 10 × dilution of Lucidea reference RNA was added to RNA from 10 000 cells, while 100 × and 400 × dilution of Lucidea test RNA was added to RNA from 1000 and 250 cells, respectively. Second strand synthesis was initiated by RNase digestion. The purified double stranded cDNA served as template for the first round of aRNA transcription. In the second round of amplification, the first strand cDNA was synthesized by priming with random hexameres. Second strand cDNA synthesis was initiated by annealing a dT/T7 primer to the aRNA/cDNA heteroduplex with partially digested aRNA. A second in vitro transcription reaction followed. The concentration of aRNA was determined by OD_260 _reading in 50 mM NaOH. An mRNA Nano Chip (Agilent Technologies, Palo Alto, CA) was used to examine the aRNA products on an Agilent 2100 Bioanalyzer.

### RNA labeling

The amounts of aRNA used in labeling reactions were; 1 *μ*g of aRNA from 10 000 cells, 0.5 *μ*g aRNA from 1000 cells, and 0.2 *μ*g aRNA from 250 cells. An indirect labeling procedure (FairPlay Microarray labeling kit, Stratagene) was performed according to the manufacturer's protocol. Random hexamers (8 *μ*g) were used to prime the cDNA synthesis. Labeled Cy5- and Cy3-cDNA was eluted from their respective columns using 50 *μ*l Tris-HCl (pH = 8.5), then mixed with 20 *μ*g of human cot-DNA (Invitrogen, Carlsbad, CA) and 300 *μ*l 0.5 × TE. The mixture was concentrated to ~10 *μ*l using Microcon YM-30 columns (Amicon, Millipore Corporation, Bedford, MA) before the addition of SlideHyb#3 hybridization buffer (Ambion Inc, Austin, TX) to a total volume of 112 *μ*l.

### Hybridization and scanning

The hybridization mixture was heated to 100°C for 3 min and subsequently centrifuged at 13 K r.p.m. for 10 min before applying to the microarray slides fitted in hybridization chambers to a GeneTac automatic hybridization station (Genomic Solutions, Ann Arbor, MI) for overnight hybridization at 65°C. Prior to scanning, the slides were washed in the following solutions: 2 × SSC and 0.1% SDS, 1 × SSC and 0.1% SDS on the hybridization stations, while two washes in 0.05 × SSC were performed manually. The slides were dried by centrifugation. Scanning was performed with an Agilent DNA Microarray Scanner, model BA (Agilent Technologies, Palo Alto, CA) at 100% laser value and variable PMT values for optimal signal acquisition. Data from the images were acquired using GenePix Pro 4.0 software (Axon Instruments Inc., Union City CA).

### Description of experiments

Three aliquots of 10 000 cells were amplified in parallel and used as reference against three amplified aliquots of 1000 and 250 cells, respectively. Hence, using a dye swap strategy, six arrays were hybridized using aRNA from replicates of 10 000 cells as a reference against aRNA from 1000 cell sample replicates. Likewise, six arrays were hybridized using 250 cell sample replicates versus reference. Thus, in total there were twelve arrays. The experimental design is presented in Fig. 1. The microarray slides were all from the same print batch.

### Data preparation

From the data for each microarray we first manually removed technically flawed spots, and secondly, we removed spots automatically flagged by the GenePix software as not found. No additional data preparation was included prior to analysis based on TransCount because this method handles even very noisy data, and normalization is incorporated into the model. For the array quality index, multiple hypothesis testing and ANOVA analysis, also spots where the spot intensity (uncorrected foreground intensity) was lower than the background intensity plus two standard deviations of the background in any of the two channels were removed. An overview of the number of filtered genes is given in Table 2. In addition, systematic errors were corrected by normalizing the data using the locally weighted scatterplot smoother, lowess, as described in Yang *et al*. [47].

### Array quality index

To ensure that the intensity in the reference channel (10 000 cell samples) did not dominate the log ratios due to differences in the amount labelled of the two comparative targets, we explored the following two parameters using filtered and normalized data. First, the SD of the log_10_-intensities of the test channel (target prepared from either 1000 cell or 250 cell samples) was calculated to give an indication of the dynamic range. Secondly, the correlation between log_10_-ratios versus the log_10_-intensities of the reference channel (10 000 cell sample) was calculated for the genes on each array to detect reference channel domination. The two parameters for each array are listed in Table 3. Scorecard genes were removed prior to this analysis.

### Multiple hypothesis testing

To identify genes with log_2_-ratios significantly different from 0, p-values were first calculated for each gene using a moderated t-test [20]. The moderated t-test applied is based on empirical Bayes analysis and is equivalent to shrinkage (or expansion) of the estimated sample variances towards a pooled estimate, resulting in a more stable inference when the number of microarray experiments is small. Separate analyses were performed for the experiments involving 10 000 cells vs. 1000 cells and 10 000 vs. 250 cells. Based on these p-values, the proportion of genes not differentially expressed was estimated using the convex decreasing density estimator of Langaas *et al*. [48]. The method is built upon the assumption that the distributions of the p-values for the genes that are not differentially expressed follow a uniform distribution. Finally, adjusted p-values were calculated using the Benjamini-Hochberg step-up procedure [49], taking the estimated proportion of genes not differentially expressed into account. Using a cut-off of the adjusted p-values at 0.05 gives an approximate level of False Discovery Rate (FDR) at 0.05.

The moderated t-test and the convex decreasing density estimator were implemented in the Limma R package available as part of the Bioconductor project [50].

### Analysis of variance modeling

To investigate the different sources of variability, we set up an ANOVA-based statistical model. Related models are found in Kerr *et al*. [51], Wolfinger *et al*. [52], Jin *et al*. [53] and Nygaard *et al*. [12]. Let log_2 _ denote the log_2_-transform of the normalized measured ratio (signal from 1000 or 250 cells divided by signal from 10 000 cells) for gene *g *on array *a*, for cell sample size *c*, for replicates *b*, *b*_*1 *_or *b*_*2*_, having dye ratio case *d*. We explain the log_2_-transformed ratio by the following model:



with *c *= 0,1, *d *= 0,1, *a *= 1,...,12, *g *= 1,...,10643, *b *= 0,1,2, *b*_*1 *_= 0,1,2, *b*_*2 *_= 0,1,2, where *μ *is the overall mean, *A*_*a *_is the overall array effect of array *a*, *C*_*c *_is the overall cell sample size effect of cell sample size *c*, *D*_*d *_is the overall dye effect of dye ratio case *d*, and *G*_*g *_is the overall gene effect of gene *g*. Furthermore, *CG*_*cg *_is the interaction between cell sample size and gene, so if this effect is significant, the cell sample size has different effect for different genes. (-1)^*d *^*DG*_*g *_represents the gene-specific dye ratio effect. *BG*_*bg *_( and  similarly) models different effect of genes for different replicates i.e. *BG*_*bg *_is the effect of gene g and replicate b of the 10 000 cell sample size. *μ*, *C*and *D *are fixed effects, the others are random effects. *B*, *B*_1 _and *B*_2 _could have been included as random effects in the model, but the number of repetitions was too small. They should therefore have been fixed effects instead. However, as they are confounded with each other and *C *and *D*, and as we expect them to be 0, they have not been included as single effects in the model. The parameters in the mixed-effects ANOVA model were estimated using Gibbs-sampling. We refer to Follestad *et al*. [54] for estimation details.

### TransCount-based absolute transcript concentration estimation

The TransCount model is based on the idea of following the mRNA molecules through the microarray experiment, from cDNA synthesis to hybridization and subsequent washing, incorporating available information about the experiment. The process is modelled as a stepwise selection, where each molecule has a certain probability of being kept in the experiment. Using a Bayesian technique, the highly multivariate joint posterior distribution of all transcript concentrations is estimated. Details about the model and estimation method are found in the article by Frigessi *et al*. [19].

The model was first applied to spikes in the reference sample (10 000 cells). Expression data from synthetic Scorecard calibration control templates pre-diluted in a serial manner by the manufacturer was incorporated into the TransCount model to obtain the respective absolute concentration measurements. We chose three spikes based on signal intensities situated clearly within the boundaries of saturation and background levels to calculate a conversion factor. This factor was obtained using linear regression and information about the known spike concentrations per cell. Furthermore, the conversion factor was used to estimate absolute transcript concentrations for each gene in the reference cell sample. These estimates represented the number of transcripts per cell before amplification. The transcript concentrations for the test samples 1000 and 250 were also estimated using the TransCount method. The results from TransCount were used to calculate the distribution of the Pearson correlation coefficients between transcript concentrations for different sample sizes. The correlation for a certain concentration c was computed from genes with concentrations in a small interval around c.

## Authors' contributions

VN performed all the laboratory work, assembled the expression data and drafted the manuscript. MH, AL and ML carried out the statistical analysis, TransCount estimations and wrote the statistical part of the manuscript. OM was in charge of the microarray production. EH conceived the study, guided the practical work, data analyses and editing of manuscript.
